# Integrated Genome-Wide Analysis of Gene Expression and DNA Copy Number Variations Highlights Stem Cell-Related Pathways in Small Cell Esophageal Carcinoma

**DOI:** 10.1155/2018/3481783

**Published:** 2018-08-30

**Authors:** Di Liu, Xinyan Xu, Junmiao Wen, Liyi Xie, Junhua Zhang, Yuxin Shen, Guoliang Jiang, Jiayan Chen, Min Fan

**Affiliations:** ^1^Department of Radiation Oncology, Fudan University Shanghai Cancer Center, Shanghai, China; ^2^Department of Radiation Oncology, The Second Military Medical University, Shanghai, China

## Abstract

*Purpose*/*Objectives*. Primary small cell esophageal carcinoma (SCEC) represents a rare and aggressive malignancy without any prospective clinical trial or established treatment strategy at present. Although previous studies have indicated similarities between SCEC and small cell lung cancer (SCLC) in terms of their clinical manifestations, pathology, and morphology, very little genetic information is available on this highly malignant tumor. At present, patients with SCEC are staged and treated according to the guidelines established for SCLC. However, early recurrence and distant metastasis are common, and long-time survivors are rare. Current options available for patients with relapsed SCEC are fairly unsatisfactory, and their prognosis is generally poor. Novel therapeutic approaches against SCEC are therefore urgently needed and require a deeper understanding of the underlying genetic mechanisms. The current investigation aims to characterize the gene expression profile and copy number variations (CNVs) in SCEC to clarify molecular markers and pathways that may possess clinical significance. *Materials*/*Methods*. De novo expression array was carried out on three matched sets of primary SCEC and adjacent normal tissue samples procured from the institutional tissue bank, utilizing the Affymetrix HG U133 Plus 2.0 Array. After individual tissue normalization, the statistical software GeneSpring GX 12.5 was used to determine differentially expressed genes (DEGs) in the tumors relative to their paired normal tissues. Gene enrichments in addition to functional annotation and gene interaction networks were performed using DAVID 6.8 and STRING 10.0, respectively. A gene alteration was determined to be recurrent if it was observed in at least 2 samples. Chromosomes X and Y were not included in calculations as gender differences are a known source of analysis bias. The DEGs of at least one SCEC sample could be mapped to the CNV regions (fold change (FC) ≥ 2 and false discovery rate (FDR) < 0.01) after gene expression profiling by RefSeq Transcript ID. These overlapped genes were subjected to the functional annotation using DAVID 6.8. In order to elucidate the effect of CNV on mRNA expression, we integrated the genome-wide copy number data and gene expression in 3 paired samples. CNV-associated gene expression aberration (CNV-FC) was calculated for the recurrent DEGs using previously published integrated microarray data as reference. Pearson's correlation coefficient was employed to determine if there was a statistical correlation between the gene expression log_2_ ratios and their copy numbers using the SPSS 19.0 software. Genes that possessed CNV-FC ≥ 2 and *r* ≥ 0.6 (*p* < 0.05) were determined to be genes potentially associated with cancer. *Results*. High-quality DNA and total RNA were first extracted from both SCEC and normal tissues. Microarray data showed significant upregulation in WNT gene sets and downregulation in the PTEN and notch gene sets in SCEC. Functional annotation showed that genes associated with DNA replication, mitosis, cell cycle, DNA repair, telomere maintenance, RB, and p53 pathways were significantly altered in SCEC compared to corresponding noncancerous tissues (Benjamini *p* < 0.05). Thirteen recurrent CNVs were found in all SCEC samples by array CGH. Chromosomal regions with gain were located in 14q11.2, and regions with loss were located in 4q22.3-23.3, 3q25.31-q29, 5p15.31-15.2, 8q21.11-24.3, and 9p23-13.1 in all samples. In two samples, the 14q11.2-32.33 region was amplified, whereas 3p26.3-25.3, 4p16.3-11, 4q11-22.3, 4q23-25, 8p23.3, and 16p13.3 were deleted. We further identified 306 genes that consistently differed in copy number and expression (194 upregulated and 112 downregulated) between the SCEC and noncancerous tissues in all three samples. These genes were significantly enriched with those involved in cell cycle, mitosis, DNA repair, P53 pathway, and RB pathway, according to their functional annotation. These 306 DEGs also included network genes of the above pathways such as NUF2, CCNE2, NFIB, ETV5, KLF5, ATAD2, NDC80, and ZWINT. In addition, 39 individual DEGs demonstrated a minimum 2-fold copy number-associated expression change (median: 5.35, 95% CI: 4.53–16.98) and Pearson's correlation coefficient ≥ 0.6 (*p* < 0.05), of which PTP4A3 showed the highest correlation (CNV-FC = 21362.13; Pearson's correlation coefficient = 0.9983; *p* = 0.037). Two distinct groups of genes belonging to each SCEC and nonmalignant tissues were observed upon unsupervised two-way (genes and samples) hierarchical clustering. *Conclusions*. The current investigation is the first to produce data regarding the genomic signature of SCEC at the transcription level and in relation to CNVs. Our preliminary data indicate possible key roles of WNT and notch signaling in SCEC and overexpressed PTP4A3 as a potential therapeutic target. Further validation of our findings is warranted.

## 1. Introduction

Primary small cell esophagus carcinoma (SCEC) is a rapidly progressive, aggressive, and rare malignancy of a specific histological type. SCEC often manifests as early lymph node invasion or distant metastases, with patients often diagnosed only when they possess advanced disease, a phenomenon that inevitably leads to dismal clinical outcomes [1, 2]. Of all the different subtypes of esophageal malignancies that occur around the world, 0.5–2.8% of these comprises of SCEC [3]. However, the incidence is as high as 7.6% in the Chinese population [4] with increasing trend. China is a region endemic for esophageal malignancies, the absolute number of SCEC patients is still high. Information is scarce surrounding its underlying pathogenesis and progression, and thus, little is known regarding potential therapeutic targets.

Although previous studies have shown clinical, pathological, and morphological similarities between SCEC and small cell lung cancer (SCLC), the genetic basis of SCEC, a highly malignant tumor, is largely unknown. Since prospective clinical trials or standard treatments have not yet been established, patients with SCEC are staged and treated according to the guidelines for SCLC. Nevertheless, early recurrence and distant metastasis is common in SCEC and long-time survival is rare. In addition, SCEC patients with relapse respond inadequately to second line of treatment making their prognosis generally poor. Therefore, the identification of novel biomarkers to aid early diagnosis, enable the design of targeted therapeutics and prognostic evaluation of SCEC, is of high clinical significance.

The initiation and development of malignancies have been well established to be triggered by the accumulation of several genetic aberrations over a long period of time [5]. DNA copy number variations (CNVs) are characteristics of a myriad of human malignancies; however, their influence on gene expression has yet to be clarified. The availability and integration of gene expression microarray data and genome-wide array-based comparative genomic hybridization (aCGH) has supplied novel insights into the molecular pathogenesis behind differing genetic expression [6–9]. An improved understanding of the underlying genetic alterations in SCEC would be the foundation of novel, innovative therapies, which are urgently needed.

In the present study, SCEC tissues were first screened for significantly altered genomic regions and differentially expressed genes (DEGs) relative to normal tissues. This was followed by an analysis where we integrated both gene copy number and expression in order to identify potential correlations between the two. Finally, quantitative reverse transcription polymerase chain reaction (qRT-PCR) analysis was performed on ten representative DEGs in order to obtain validated results obtained via microarray analysis.

## 2. Materials and Methods

### 2.1. Tissue Processing, Genomic DNA, and Total RNA Extraction

Paired SCEC and adjacent noncancerous tissues from three surgical specimens were harvested surgically and frozen with liquid nitrogen, before being kept at −80°C in the tissue bank of Fudan University Shanghai Cancer Center (FUSCC). All three patients had middle thoracic SCEC stage III (based on the American Joint Committee on Cancer (6th edition) TNM staging system) or limited stage (based on the Veteran's Administration Lung Cancer Study Group, VALSG). The median age of all patients was 59 years (ranges from 56 to 67), with two of the three patients being female. All selected patients had not received any anticancer therapy before surgery nor were they afflicted with any other cancer type. Ethical approval was granted by the Human Research Ethics Committee of FUSCC prior to commencement of this study, and informed consent was procured from all patients before enrollment.

Frozen tissue blocks were first sectioned and subjected to cresyl violet or toluidine blue staining to visualize total RNA and genomic DNA (gDNA), respectively (Ambion, Austin, TX, USA). LMD (Leica Microsystems, Wetzlar, Germany) was used by pathologists to discern between malignant and nonmalignant cells in the tissue sections. Total RNA and gDNA were extracted with the help of commercially available kits and were performed based on to the manufacturer's instructions. Agilent's 2100 Bioanalyzer (Agilent Technologies, Palo Alto, CA) was used to confirm the purity, integrity, and concentration of total RNA (data not shown). RNA integrity was determined with the RIN software algorithm [10], and only samples with a RIN score of >7.5 were used for microarray experiments. High-quality RNA characteristics were samples that possessed low background noise and had distinct peaks representing 18S and 28S ribosomal RNA. 1% agarose gel electrophoresis was used to verify gDNA quality, while its concentration was quantified with a Nanodrop ND-1000 spectrophotometer (Nanodrop Technologies, Wilmington, DE).

### 2.2. Array CGH

Genomic abnormalities in the SCEC tissues were analyzed by Agilent aCGH G3 Human 4x180k Array. The gDNA (500 ng) of cancerous and noncancerous samples was digested overnight at 37°C using the Rsa I and Alu I restriction site enzymes (Promega, Madison, WI). Cy5-/Cy3-dUTP fluorescent dyes were used to label both samples with the Agilent Genomic DNA Labeling Kit Plus (Agilent Technologies). The labeled gDNA products were purified with Microcon YM-30 filtration device (Millipore, Bedford, MA), and the yield of DNA and dye incorporation were quantified. CGH microarrays were used to hybridized mixtures of the labeled sample pairs (containing identical quantities of each malignant and nonmalignant samples) at 65°C for 24 hours. The slides were then rinsed with Agilent Oligo aCGH Wash Buffer 1 and 2 (Thermo Shandon, Waltham, MA, US) as per the manufacturer's instructions. Following washing, an Agilent microarray scanner was used to scan the slides. The Feature Extraction software version 10.7 was used to analyze raw data at the default CGH parameter settings (Agilent Technologies).

Array CGH data was processed as previously described. Briefly, putative CNV intervals contained in each sample were identified using CytoGenomics 2.7.8.0 (Agilent Technologies, Santa Clara, CA, US). Conversion of the Cy5/Cy3 ratios into log_2_-transformed values was then carried out with data corrections carried out using fuzzy zero and centralization corrections. Lastly, the ADM-2 algorithm was used to identify CNVs in individual SCEC samples at threshold 6 before proceeding to locate aberration frequencies. Additionally, the following aberration filters were applied: maximum number of aberration regions = 10000, minimum absolute average log_2_ ratio for region = 0.5, and minimum number of probes in region = 3. The analysis excluded chromosomes X and Y. Data on the original copy number was submitted to the NCBI's Gene Expression Omnibus (GEO) [11] and is accessible through the GEO Series accession number GSE111298. A recurrent variation was determined to be present if recurrence was observed in at least 2 of the 3 samples. Minimum common regions of recurrent variations in the 3 samples were analyzed, including the chromosomal positions and size of the aberrations.

### 2.3. Gene Expression Microarray Analysis

Gene expression profiling of the SCEC and noncancerous tissues was carried out with the Affymetrix HG U133 Plus 2.0 Array (Affymetrix, Santa Clara, CA). The GeneChip 3'IVT Express Kit (Affymetrix, Santa Clara, CA, US) was used to amplify, label, and purify total RNAs as per the manufacturer's protocols. Biotin-labeled cRNA was hybridized at 45°C for 16 hours, and the gene chips were rinsed and stained with streptavidin-phycoerythrin (Molecular Probes) with the GeneChip Fluidics Workstation 450 (Affymetrix). A confocal laser scanner (GeneArray Scanner 3000) was utilized to scan the stained gene chips, with the resultant images converted by the Command Console software 3.1 (Affymetrix) at default settings into corresponding numerical values that indicated their relative signal intensities. Raw data were normalized by robust multiarray average (RMA) quantile normalization analysis algorithm with the GeneSpring GX 12.5 software (Agilent Technologies, Santa Clara, CA, US) to generate CEL intensity files. All data on gene expression can be viewed at NCBI via GEO (GEO: GSE111044).

Quality control was performed by the following diagnostic plots: principal component analysis (PCA), boxplots, Pearson's correlation, and MvA plots, (Supplementary information Figure S1, S2). Moderated *t*-test analysis with Benjamini-Hochberg multiple testing correction was used to obtain DEGs whose fold change (FC) between SCEC and matched adjacent noncancerous tissues was ≥2 (with a false discovery rate (FDR) cut-off < 0.01). DEGs were visualized in volcano plots (Supplementary information Figure S3) and then imported to gene set enrichment analysis (GSEA) version 2.2.3 software to interpret the gene expression data. The focus was on oncogenic signatures gene sets (C6), rather than individual genes, that share common biological functions. Functional annotation and gene interaction networks of the enriched genes were analyzed by DAVID 6.8 and STRING 10.0, respectively.

### 2.4. Integrative Analysis of the aCGH and Expression Data

To locate genes whose expression was influenced by genomic CNVs, DEGs with FDR < 0.01 and FC ≥ 2 that were located in CNV regions of at least one SCEC sample were tracked using the RefSeq Transcript ID. These overlapping genes were subjected to functional annotation using DAVID 6.8. Deletions and amplifications were regarded as separate entities during the analysis.

Pearson's correlation coefficients were also calculated to determine potential correlations between gene expression and DNA copy number. Only genes located in chromosomal regions that possessed recurrent aberrations were analyzed. By determining DEGs that were associated with an abnormal DNA copy number, we sought to isolate stability genes, tumor suppressor genes, and potential oncogenes that carried out mechanistic functions in cancers. Gene median expression levels between samples with and without copy number deletions/amplifications were contrasted to determine the impact that copy number differences had on the expression of genes. Gene expression fold changes (FCs) were calculated by dividing the median expression in sample(s) with CNVs by the median expression in sample(s) without CNVs [6, 12]. Genes that were identified were those that possessed a minimum 2-fold copy number increase and an associated gene expression aberration (CNV-FC). We expected to find these genes to be expressed differentially between SCEC and normal esophageal tissues. This hypothesis was proved by hierarchical clustering of the 3 sample pairs using the average linkage method, and the clusters were visualized using the Java TreeViewer 1.1.3 software. With the help of the SPSS 19.0 software, we were able to determine the Pearson's correlation coefficients between DNA CNVs and changes in expression level for each selected gene in order to clarify the association between copy number and gene expression. Genes that possessed CNV-FC ≥ 2 and *r* ≥ 0.6 (*p* < 0.05) were determined to be genes potentially associated with cancer.

### 2.5. Quantitative Reverse Transcription Polymerase Chain Reaction (qRT-PCR)

qRT-PCR was performed to verify DEGs determined in through microarray analysis. Briefly, TRIzol reagent (Invitrogen) was used to extract total tissue sample RNA followed by cDNA synthesis utilizing PrimeScript RT reagents (Takara Bio Inc.). Using SYBR Green dye (Takara Bio Inc.), gene expression levels were determined on a 7500 Fast Real-Time PCR cycler (Applied Biosystems). Specific gene primers were designed and constructed by BioTNT Co. (Shanghai, China). All primer sequences are available in Table S1 (Supplementary information). All reactions were carried out in triplicates. The 2^−ΔΔCt^ method was used to determine relative gene expressions, normalized to the expression of housekeeping gene *β*-actin.

## 3. Results

### 3.1. Gene Expression Profile of SCEC

Data analysis through GeneSpring software revealed a total of 1485 DEGs in SCEC relative to the adjacent noncancerous tissues, with 879 upregulated and 606 downregulated genes. Among the 1485 DEGs, neuroendocrine-associated genes SYP (Syn; FC = 1.5, FDR = 0.04), CHGA (CgA; FC = 3.02, FDR = 0.04), NCAM1 (CD56; FC = 18.10, FDR = 0.006), ASCL1 (FC = 619.23, FDR = 0.0005), and GRP (FC = 5.33, FDR = 0.03) and proliferation-associated genes MKI67 (Ki-67; FC = 9.35, FDR = 0.007) and PCNA (FC = 4.20, FDR = 0.006) were overexpressed. GSEA C6 annotation identified that PTEN-, RB-, and WNT-related gene sets were upregulated while Notch-related gene sets were downregulated (Table 1).  Table 2 lists the biological processes or pathways of the genes as annotated by DAVID based on their significance (count ≥ 10 and Benjamini *p* < 0.01), including DNA replication, cell cycle, mitosis, telomere maintenance, DNA repair, p53, and RB. Furthermore, SCEC tissues were found to possess interactive gene networks with FOXM1, TMPO, KIF11, NEK2, and CENPF as common skeleton centered on NUF2 (Supplementary information Figure S4). The genes involved in the SCEC-regulated network were involved in cell cycle, mitosis, cell cycle checkpoints, spindle organization, microtubule binding, cytoskeletal protein binding, and other biological processes (Supplementary information Table S2, S3).

### 3.2. Copy Number Variations in SCEC

CNVs were found to be expressed across the entire genome based on analyses of the mean frequencies of copy number gains and losses. Supplementary information Figure S5 depicts CNVs found across all chromosomes. The gained regions that were detected in all samples were located in 14q11.2, whereas the lost regions detected in all samples were located at 4q22.3-23.3. Regions of gain observed in 2 samples were located at 3q25.31-q29, 5p15.31-15.2, 8q21.11-24.3, 9p23-13.1, and 14q11.2-32.33, and regions of loss observed in 2 samples were located at 3p26.3-25.3, 4p16.3-11, 4q11-22.3, 4q23-25, 8p23.3, and 16p13.3 in decreasing order of frequency. Minimal common regions of these altered copy numbers, including the chromosomal position, potential target genes, frequency, and size of the base pair alterations, are shown in Table 3. Only genes that possessed a minimum twofold copy number along with associated variations in levels of gene expression and also a Pearson's correlation coefficient of less than 0.6 (*p* < 0.05) were selected.

### 3.3. Copy Number-Associated Gene Expression Changes

We identified 306 genes (194 upregulated and 112 downregulated) that consistently showed a change in copy number as well as expression levels. These genes were significantly enriched in the cell cycle, mitosis, DNA repair, p53 pathway, and RB pathways, according to the functional annotation (Supplementary information Table S4). Notably, most of the network genes in the gene expression profiling, such as NUF2, CCNE2, NFIB, ETV5, KLF5, ATAD2, NDC80, and ZWINT, were included in these 306 genes.

**Figure 1:**
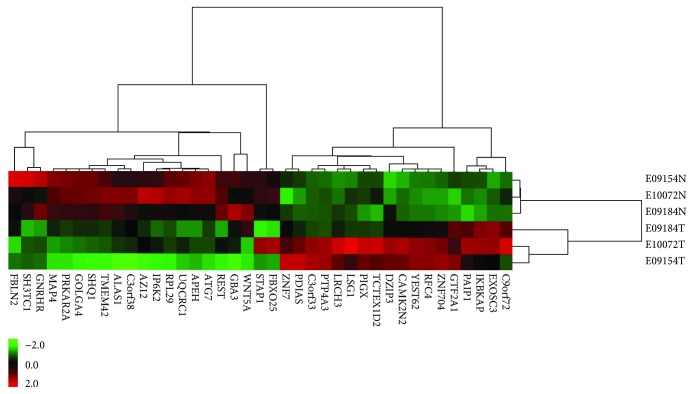
A 39-gene unsupervised hierarchical clustering of 3 pairs of SCEC samples uncovered two distinct clusters separating SCEC samples from adjacent noncancerous samples. Underexpressions are denoted in green while overexpressions are denoted in red.

Thirty-nine individual genes had both a minimum 2-fold copy number-associated change in expression (median: 5.35, 95% CI: 4.53–16.98) and Pearson's correlation coefficient of less than 0.6 (*p* < 0.05; see Supplementary information Table S5 for details), and PTP4A3 showed the highest correlation (CNV-FC = 21362.13; Pearson's correlation coefficient = 0.9983; *p* = 0.037). An unsupervised two-way (genes and samples) hierarchical clustering of the 3 sample pairs based on these genes revealed two distinct clusters that separated the SCEC from adjacent noncancerous tissues (Figure 1). Several novel genes that may serve as SCEC biomarkers were revealed during an integrated analysis of gene copy number and expression; however, their clinical utility needs to be verified through further studies.

### 3.4. qRT-PCR Validation of Microarray Results

**Figure 2:**
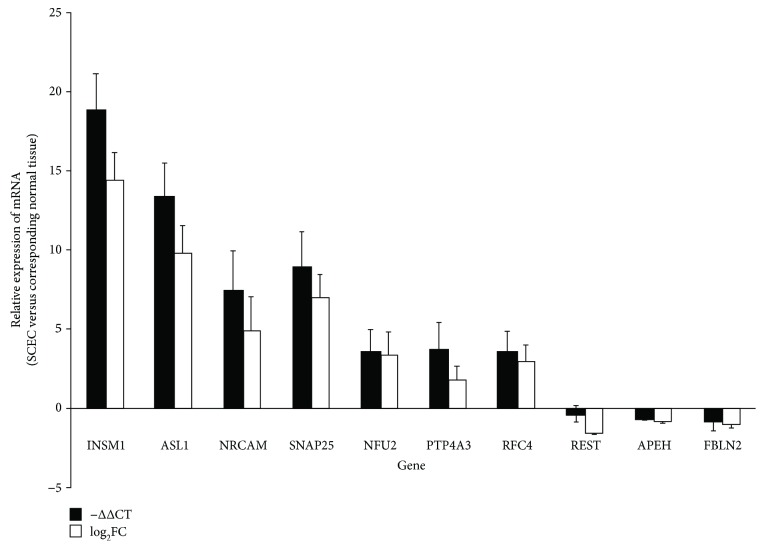
The mRNA level of *INSM1*, *ASCL1*, *NRCAM*, *SNAP25*, *NUF2*, *PTP4A3*, *RFC4*, *REST*, *APEH*, and *FBLN2* in SCEC. Expression levels in SCEC were compared with the corresponding normal tissues. The *x*-axis displays gene symbols, and the *y*-axis depicts gene expression log ratios derived from qRT-PCR or microarray. Bars: standard error (SE).

To substantiate the microarray results, qRT-PCR was performed on the following 10 out of the 39 genes: neuroendocrine-associated genes (INSM1, ASCL1, NRCAM, and SNAP25), one gene centered in the gene regulatory network (NUF2), and 5 possible cancer-associated genes (PTP4A3, RFC4, REST, APEH, and FBLN2). Seven of the 10 genes, that is, INSM1, ASCL1, NRCAM, SNAP25, NUF2, PTP4A3, and RFC4, were upregulated while REST, APEH, and FBLN2 were downregulated. The qRT-PCR results mirrored those obtained via high-throughput microarray analysis, thus validating the latter as well as highlighting some potential target genes (Figure 2).

## 4. Discussion

There are currently no effective therapeutic strategies for treating primary SCEC, a rare malignancy. Unfortunately, no major progress has been made in the last decades to elucidate the biology of SCEC. This study is the first to study the molecular and genetic basis of SCEC on a genome-wide level. Compared to previous studies, we have discovered more genes through global microarray analysis that may have a potential role in the mechanism of SCEC. The ability of a malignant tumor to proliferate is a feature of great prognostic value in the clinical setting [13]. Malignant SCEC cells have the propensity to multiply quickly, with patients rapidly developing hematogenous, bone, and lymph node metastasis early in the course of the disease. Consistent with this, and in line with previously published reports [14], we saw a significant upregulation in neuroendocrine-associated and proliferation-associated genes in SCEC tissues relative to corresponding normal tissues.

Although promalignant features like high levels of ki-67, proliferating cell nuclear antigen (PCNA) and telomerase activity, Bcl-2 positivity, rich neovascularization, and p53 overexpression have been reported in SCEC [14–16], detailed genetic studies are not available. PTEN was the most significantly altered (downregulated) gene in our genome-wide analysis of SCEC tissues. It is an effector of the PI3K/PTEN/AKT pathway; a critical pathway that regulates cell cycle progression, cell migration, metabolism, and survival. Furthermore, aberrant PTEN expression brought about either via promotor methylation silencing, deletions, or mutations is often commonly observed in several primary and metastatic human cancers. There appears to be a higher occurrence of mutations in the PTEN gene (36.84%) in patients of Chinese ethnicity with SCEC in contrast to EGFR, KARS, or PIK3CA mutations [4]. In addition, PTEN is often lost or mutated in SCLC [17, 18]. Taken together, PTEN presents itself as a potential therapeutic target for SCEC.

We also performed a genome-wide analysis of DNA CNVs in SCEC tissues to determine genes that possessed dysregulated expression levels as a result of altered copy numbers. 7 chromosomal regions were observed to have recurrent copy number losses, while 6 chromosomal regions demonstrated recurrent copy number gains. This highlights that these CNVs, in addition to the specific genes, may have a significant biological role in SCEC pathogenesis. We identified a total of 306 consistent DEGs that were significantly enriched in cell cycle, mitosis, DNA repair, p53, and RB pathways as per functional annotation.

To further highlight the association between the expression of genes located in chromosomes with recurrent aberration expression and copy number, we calculated their Pearson's correlation coefficient. Thirty-nine genes were found to possess an *r* value of ≥0.6, indicating that their expression fold changes correlated in a statistically significant manner to their copy number. The highest correlation was shown by PTP4A3 (CNV-FC = 21362.13; *r* = 0.9983; *p* = 0.037). PTP4A3, also known as phosphatase of regenerating liver-3 (PRL-3), is a protein tyrosine phosphatase closely related to metastasis, with its expression level found to correlate significantly with the survival and progression of a myriad of cancerous tumors. A recent study has documented that PRL-3 may adversely affect tumor development by mediating deleterious effects on telomere homeostasis [19]. Based on previous findings and the exceptionally aggressive nature of SCEC, we hypothesize that PTP4A3, with its key roles in SCEC genesis, development, and metastasis, may serve as a target for the development of therapeutic agents.

Although we only examined a small number of the primary samples, our study is the first to examine gene expression profiles and CNVs in SCEC patients on a genome-wide scale. Further studies are needed on larger sample cohorts to validate our findings and to single out the most useful genes. Furthermore, the clinical and therapeutic significance of PTP4A3 and other potential targets has to be validated. Taken together, our study is an important step in elucidating the mechanistic basis of SCEC genesis and metastasis and discovering novel therapeutic targets.

## 5. Conclusion

This study was the first to investigate the genomic signature of SCEC from genome-wide expression and copy number analysis. Our preliminary data indicates that stem cell-related genes and pathways might function to mediate the initiation, development, and metastasis of SCEC, although further validation is warranted.

## Figures and Tables

**Table 1:** Oncogenic signatures gene sets significantly altered in SCEC.

Name	Size	NES	NOM *p* val	FDR *q* val
PTEN_DN.V2_UP	10	2.06	0.00252	0.0270
RB_P107_DN.V1_DN	13	2.04	0.00278	0.0264
RB_P130_DN.V1_DN	11	2.01	0.00270	0.0272
KRAS.300_UP.V1_DN	14	1.98	0.00882	0.0285
WNT_UP.V1_DN	13	1.84	0.0158	0.0580
IL2_UP.V1_UP	21	1.81	0	0.0630
IL15_UP.V1_UP	19	1.56	0.0262	0.221
KRAS.600.LUNG.BREAST_UP.V1_DN	27	1.55	0.0350	0.212
NOTCH_DN.V1_UP	18	1.51	0.0327	0.191
MEK_UP.V1_UP	28	1.50	0.0464	0.234
ERB2_UP.V1_UP	25	1.49	0.0425	0.207

Differentially expressed genes (DEGs) were annotated by gene set enrichment analysis (GSEA). Threshold values: size ≥ 10 and NOM *p* val < 0.05. FDR = false discover rate; NOM *p* val = nominal *p* value; NES = normalized enrichment score.

**Table 2:** DAVID annotation of DEGs in SCEC group.

Database	Name	Count	Benjamini *p* value
KEGG	*DNA replication*	19	8.88*E* − 10
	*Cell cycle*	33	8.61*E* − 09
	*P53 signaling pathway*	19	4.80*E* − 05
	Progesterone-mediated oocyte maturation	18	0.00457
	Base excision repair	11	0.00419
	Oocyte meiosis	20	0.00790
REACTOME	*Cell cycle, mitotic*	100	3.47*E* − 36
	*DNA replication*	29	7.19*E* − 08
	*DNA repair*	22	0.00173
	Cell cycle checkpoints	23	0.00184
	*Telomere maintenance*	14	0.00713
PANTHER	*P53 pathway*	22	0.0456
GO BP (TOP10)	*M phase*	99	3.91*E* − 28
	*M phase of mitotic cell cycle*	78	1.97*E* − 26
	*Mitosis*	77	2.12*E* − 26
	*DNA replication*	61	2.41*E* − 18
	*DNA metabolic process*	98	2.54*E* − 13
	Mitotic sister chromatid segregation	18	1.73*E* − 07
	Spindle organization	20	1.55*E* − 07
	Cell cycle checkpoint	27	2.09*E* − 06
	Regulation of cell cycle process	28	6.97*E* − 05
	*DNA repair*	50	7.34*E* − 05
GO MF	*Pyrophosphatase activity*	91	0.00138
	*Adenyl ribonucleotide binding*	152	0.00573
	*Guanyl ribonucleotide binding*	44	0.0486

Threshold values: count ≥ 10 and Benjamini *p* value < 0.01. The biological processes or pathways in common between SCEC and SCLC are in italics. BP = biological process; GO = gene ontology; MF = molecular function.

**Table 3:** Minimal common regions of recurrent copy number amplification and deletion (*n* ≥ 2).

Chromosomal aberration	Positon (Mb)	Size (Mb)	*n* = 3 (%)	Samples	Possible cancer-associated genes
Gains					
14q11.2	20.22–23.91	3.69	3 (100%)	1, 2, 3	—
3q25.31-q29	155.59–197.83	42.25	2 (67%)	1, 3	*TCTEX1D2*, *YEATS2*, *PIGX*, *LRCH3*, *RFC4*, *LSG1*, *CAMK2N2*
5p15.31-15.2	8.87–10.99	2.12	2 (67%)	1, 2	*PAIP1*
8q21.11-24.3	74.51–146.28	71.76	2 (67%)	1, 3	*ZNF704*, *PTP4A3*, *ZNF7*
9p23-13.1	9.38–39.07	29.7	2 (67%)	1, 2	*EXOSC3*
14q11.2-32.33	19.38–20.22	0.84	2 (67%)	1, 2	—
	23.91–107.29	83.37	2 (67%)	1, 3	—
Losses					
4q22.3-23	98.14–99.10	0.96	3 (100%)	1, 2, 3	—
3p26.3-25.3	0.07–90.25	90.07	2 (67%)	1, 2	*IP6K2*, *APEH*, *TMEM42*, *WNT5A*, *UQCRC1*, *C3orf38*, *RPL29*, *PRKAR2A*, *MAP4*, *AZI2*, *ALAS1*, *GOLGA4*, *FBLN2*, *SHQ1*, *ATG7*
4p16.3-11	0.72–49.06	48.99	2 (67%)	2, 3	*GBA3*, *SH3TC1*
4q11-22.3	52.69–98.14	45.45	2 (67%)	2, 3	*STAP1*, *GNRHR*, *REST*
4q23-25	99.10–107.92	8.22	2 (67%)	2, 3	
8p23.3	0.18–0.50	0.33	2 (67%)	2, 3	—
16p13.3	5.50–5.61	0.12	2 (67%)	1, 3	—
	6.75–6.82	0.06	2 (67%)	1, 3	—

## Data Availability

The microarray data used to support the findings of this study have been deposited in the Gene Expression Omnibus (GEO). The GEO accession numbers are appended as follows: GSE111299: genome-wide analysis of gene expression and DNA copy number variations in small cell esophageal carcinoma, GSE111044: expression data from SCEC and corresponding normal samples, and GSE111298: aCGH data from SCEC and corresponding normal samples. Websites included are https://www.ncbi.nlm.nih.gov/geo/query/acc.cgi?acc=GSE111044 and https://www.ncbi.nlm.nih.gov/geo/query/acc.cgi?acc=GSE111298.

## Supplementary Materials

Supplementary Materials Figure S1: PCA, box plot, and Pearson's correlation matrix of SCEC group. Figure S2: MvA plot of each SCEC samples and adjacent normal tissues. Figure S3: volcano plots of SCEC groups. Figure S4: gene regulatory network plotted by the top 120 DEGs (ranked by FDR) of SCEC group using STRING 10.0.5. DNA copy number change profiles of 3 pairs of SCEC samples. Table S1: primers utilized in the qRT-PCR validation of gene expression microarray data. Table S2: list of regulatory network genes in SCEC group. Table S3: DAVID annotation of regulatory network genes in SCEC group. Table S4: DAVID annotation of genes changed consistently between mRNA expression and copy number in SCEC. Table S5: list of 39 possible cancer-associated genes in SCEC.
