# Supplementary material for: Integrated Genome-Wide Analysis of Gene Expression and DNA Copy Number Variations Highlights Stem Cell-Related Pathways in Small Cell Esophageal Carcinoma
Source: Stem Cells Int. 2018 Aug 30;2018:3481783. doi: 10.1155/2018/3481783 (PMC6136462; doi:10.1155/2018/3481783)
Supplement: Supplementary Materials — Figure S1: PCA, box plot, and Pearson's correlation matrix of SCEC group. Figure S2: MvA plot of each SCEC samples and adjacent normal tissues. Figure S3: volcano plots of SCEC groups. Figure S4: gene regulatory network plotted by the top 120 DEGs (ranked by FDR) of SCEC group using STRING 10.0.5. DNA copy number change profiles of 3 pairs of SCEC samples. Table S1: primers utilized in the qRT-PCR validation of gene expression microarray data. Table S2: list of regulatory network genes in SCEC group. Table S3: DAVID annotation of regulatory network genes in SCEC group. Table S4: DAVID annotation of genes changed consistently between mRNA expression and copy number in SCEC. Table S5: list of 39 possible cancer-associated genes in SCEC. [file 3481783.f1.pdf]

Lists of figures

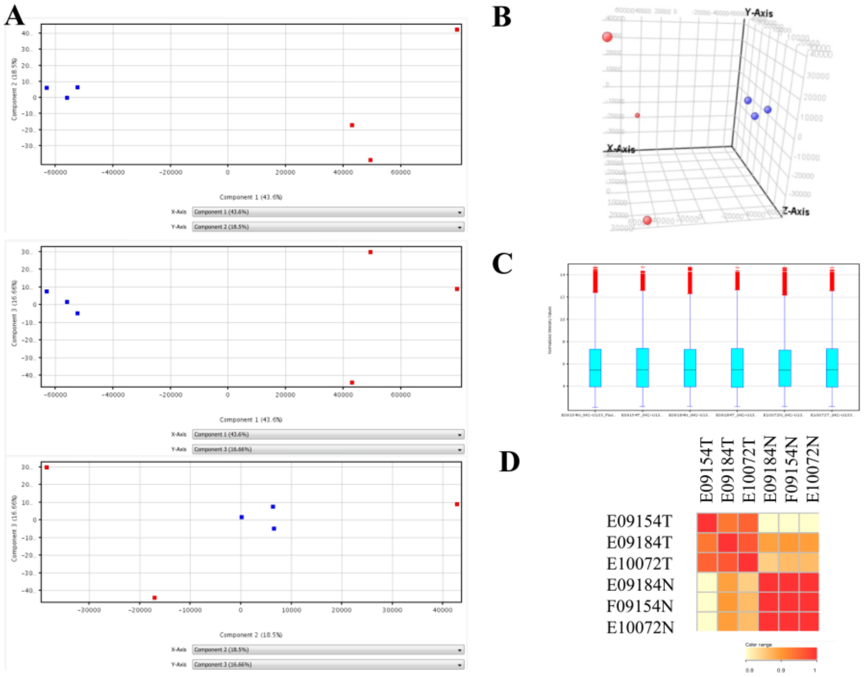

Figure S1: A-B: Principal Component Analysis (PCA) was applied to 3 pairs of SCEC tissues (red) and matched adjacent normal tissues (blue) that were characterized by the gene expression of all probes on Affymetrix HG U133 Plus 2.0 Array. **C:** Box plot of SCEC group. **D:** Pearson's correlation matrix of SCEC group.

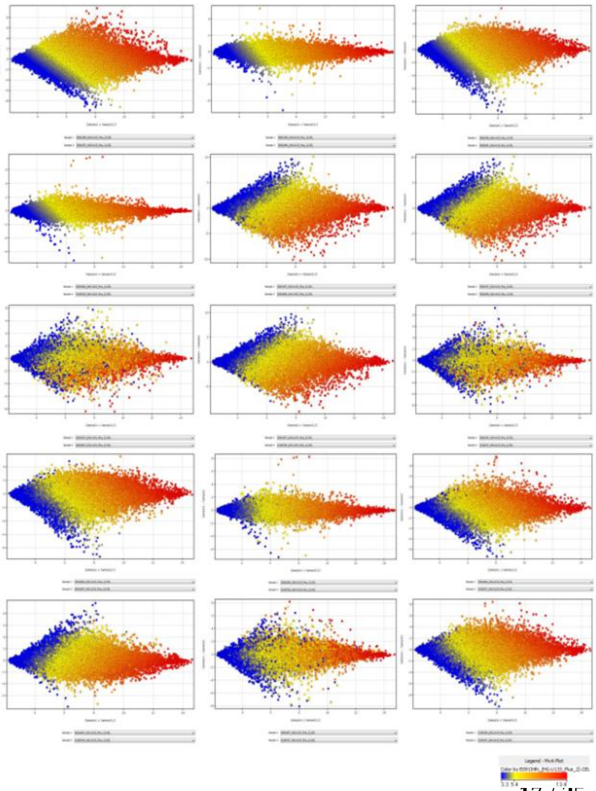

Figure S2: MvA plot of SCEC group.

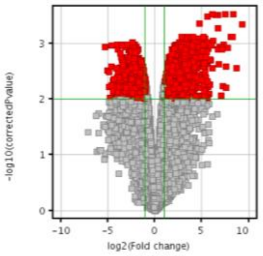

Figure S3: Volcano plots of probe sets differing between SCEC and matched normal tissues. Fold change (X axis) is plotted against statistical significance (Y axis) for each probe sets. Genes altered with a fold change ≥2 and FDR <0.01 are depicted in red. Grey represents genes in the arrays that were not found to differ significantly between cancerous samples and matched normal samples.

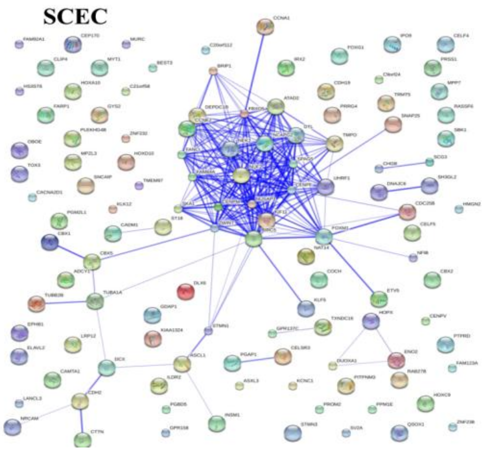

Figure S4: Gene regulatory network plotted by the top 120 DEGs (ranked by FDR) of SCEC groups.

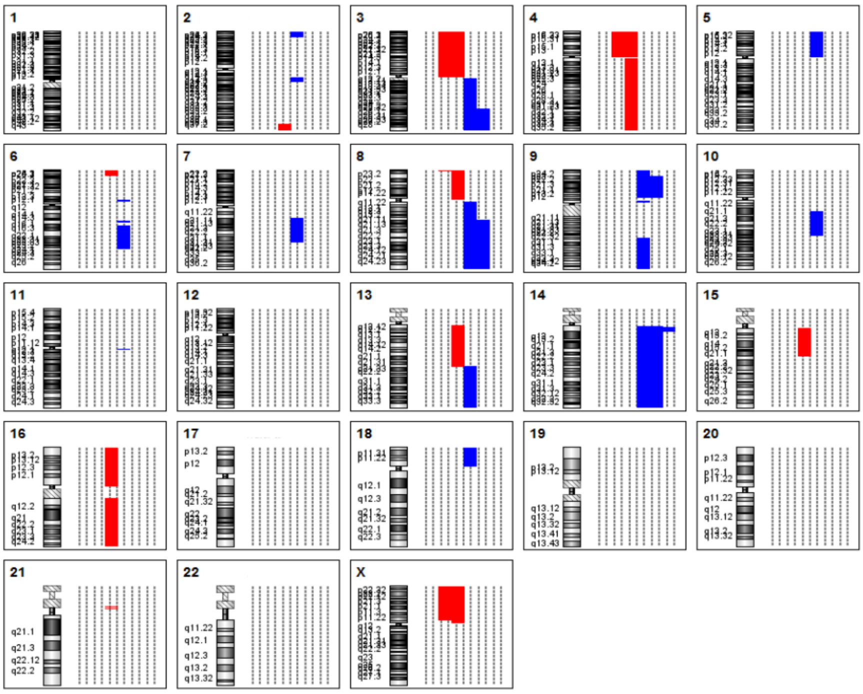

Figure S5: DNA copy number change profiles in 3 pairs of SCEC samples. The CNVs frequency of the whole genome was analyzed by aCGH. Gains were marked in red and losses in bule.

Lists of tables

Table S1. Primers used in qRT-PCR for microarray gene expression validation

Gene	Forward Primer (5’-3’)	Reverse Primer (5’-3’)	Product
β-actin	AAGGTGACAGCAGTCGGTT	TGTGTGGACTTGGGAGAGG	195bp
INSM1	GTATTCGCTGTGTTCATGGTC	CGCTACATACATAGAGAGCAGAG	79bp
ASCL1	AACTCCCATCACCTCTAACA	TGAGACGAAAGACACCAACT	120bp
NRCAM	GATGGCGAAGAATGAAGTT	ACAGTGAGGGATAAGGTGTG	141bp
NUF2	ATGATGCCAGTGAACTCTGAA	GACTTGTCCGTTTTGCTTTTG	160bp

SNAP25	CCTGGATATGGGCAATGAGAT	ACACGGGTGGGCACACTTA	146bp
PTP4A3	GCTTCCTCATCACCCACAA	CCGTACTTCTTCAGGTCCTCA	70bp
RFC4	CACCCGATTCTGTCTTATCTG	TCCTTCTTGGCAATGTCTAGT	135bp
REST	TTTAAGAGAAGAGGCATCAG	GATATTAGCAGCAAGACCAG	127bp
APEH	GTATTACCGTGCCCTCAAGAC	AAGTGTGTGCGTAGCCAGA	135bp
FBLN2	AGACCCCAACTCTGTCCATTC	CTCCAGGCACTCGTCATTGT	121bp

Table S2. List of regulatory network genes in SCEC group

Gene symbol	Gene title	Probe set	FC	*p* value	FDR
INSM1	Insulinoma-associated 1	206502_s_at	321	1.60E-08	2.91E-04
ASCL1	Achaete-scute complex homolog 1	209988_s_at	619	7.48E-08	4.55E-04
TUBB2B	Tubulin, beta 2B class IIb	214023_x_at	102	9.67E-08	5.29E-04
NUSAP1	Nucleolar and spindle associated protein 1	219978_s_at	13.1	2.14E-07	7.65E-04
DCX	Doublecortin	204851_s_at	261	2.47E-07	7.65E-04
NRCAM	Neuronal cell adhesion molecule	204105_s_at	24.8	3.26E-07	8.11E-04
SNAP25	Synaptosomal-associated protein	202508_s_at	74.7	3.79E-07	9.01E-04
CCNE2	Cyclin E2	205034_at	13.0	4.46E-07	9.39E-04
CENPF	Centromere protein F	207331_at	7.44	5.25E-07	9.49E-04
CCNA1	Cyclin A1	205899_at	17.3	4.98E-07	9.49E-04
TUBA1A	Tubulin, alpha 1a	209118_s_at	6.12	5.38E-07	9.49E-04
NUF2	NDC80 kinetochore complex component, homolog	223381_at	11.7	1.38E-06	1.06E-03
FAM64A	Family with sequence similarity 64, member A	221591_s_at	9.13	1.30E-06	1.06E-03
NEK2	NIMA (never in mitosis gene a)-related kinase 2	204641_at	10.1	9.34E-07	1.06E-03
SKA1	Spindle and kinetochore associated complex subunit 1	217640_x_at	5.34	6.67E-07	1.06E-03
SPAG5	Sperm associated antigen 5	203145_at	9.23	6.83E-07	1.06E-03
TMPO	Thymopoietin	209754_s_at	8.36	9.86E-07	1.06E-03
CDC25B	Cell division cycle 25 homolog B	201853_s_at	5.53	1.16E-06	1.06E-03
NFIB	Nuclear factor I/B	209289_at	4.76	1.18E-06	1.06E-03
CBX1	Chromobox homolog 1	201518_at	4.28	1.46E-06	1.06E-03
CDH2	Cadherin 2, type 1, N-cadherin	203440_at	50.0	1.54E-06	1.07E-03
ETV5	Ets variant 5	203348_s_at	4.93	1.61E-06	1.07E-03
FBXO5	F-box protein 5	218875_s_at	8.89	1.65E-06	1.07E-03
DTL	Denticleless E3 ubiquitin protein ligase homolog	222680_s_at	10.6	1.63E-06	1.07E-03
KIF11	Kinesin family member 11	204444_at	6.79	1.79E-06	1.07E-03
KLF5	Kruppel-like factor 5	209211_at	-4.07	1.97E-06	1.13E-03

FANCI	Fanconi anemia, complementation group I	213007_at	5.58	1.98E-06	1.13E-03
STMN1	Stathmin 1	200783_s_at	11.7	2.59E-06	1.15E-03
CTTN	Cortactin	214073_at	-5.24	2.29E-06	1.15E-03
FOXM1	Forkhead box M1	202580_x_at	8.26	2.47E-06	1.15E-03
ZWINT	ZW10 interactor	204026_s_at	6.47	2.68E-06	1.16E-03
CBX5	Chromobox homolog 5	231862_at	4.95	2.73E-06	1.17E-03
CENPM	Centromere protein M	218741_at	4.58	2.88E-06	1.20E-03
NCAPG2	Non-SMC condensin II complex, subunit G2	219588_s_at	5.19	3.01E-06	1.21E-03
ATAD2	ATPase family, AAA domain containing 2	228401_at	6.57	3.11E-06	1.21E-03
UHRF1	Ubiquitin-like with PHD and ring finger domains 1	225655_at	10.7	3.22E-06	1.23E-03
DEPDC1B	DEP domain containing 1B	226980_at	11.9	3.31E-06	1.24E-03
BRIP1	BRCA1 interacting protein C-terminal helicase 1	235609_at	7.85	3.56E-06	1.25E-03
BIRC5	Baculoviral IAP repeat containing 5	202095_s_at	7.13	3.63E-06	1.25E-03

Abbreviations: FC= fold change; FDR= false discover rate.

Table S3. DAVID annotation of regulatory network genes in SCEC group

Database	Name	Count	Benjamini *p* value
REACTOME	Cell Cycle, Mitotic	12	3.47E-36
GO BP5	Mitosis	14	3.82E-12
	M phase of mitotic cell cycle	14	2.42E-12
	M phase	15	9.29E-12
	Spindle organization	5	5.12E-04
	Regulation of mitotic cell cycle	6	0.00352
	Cell cycle checkpoint	5	0.00556
GO MF5	Tubulin binding	5	0.00731
	Microtubule binding	5	0.0287
	Cytoskeletal protein binding	7	0.0262

Abbreviations: GO= gene ontology; BP= biological process; MF= molecular function.

Table S4. DAVID annotation of genes changed consistently between copy number and mRNA expression in SCEC

Database	Name	Count	*p* value
KEGG	**DNA replication**	6	1.42E-04
	**P53 signaling pathway**	6	0.00274
	**Cell cycle**	7	0.00868
BBID	**RBphosphoE2F**	5	0.0195
REACTOME	**Cell Cycle, Mitotic**	17	1.34E-05
	**DNA Repair**	7	0.00556

	**Cell Cycle Checkpoints**	7	0.00872
	**DNA Replication**	6	0.0193
PANTHER	**P53 pathway**	6	0.0269
GO BP	**DNA replication**	15	8.85E-06
(TOP10)	**DNA metabolic process**	24	4.43E-05
	Mitotic sister chromatid segregation	6	4.39E-04
	**DNA repair**	14	0.00197
	**Mitosis**	12	0.00219
	**M phase of mitotic cell cycle**	12	0.00251
	DNA replication initiation	5	0.00274
	**Cell cycle checkpoint**	7	0.00604
	**M phase**	14	0.00682
GO MF	Ras guanyl-nucleotide exchange factor activity	7	0.00278
	**Adenyl ribonucleotide binding**	32	0.0457

[a] DAVID annotation results of genes changed consistently between copy number and mRNA expression were highly concordant with biological processes or pathways annotated by DEGs in SCEC groups (in bold).

Table S5. List of 39 possibly cancer-associated genes in SCEC

Gene symbol	Gene title	Cytoband	Re-gulation	CNV-FC	Pearson r	*p* value
GTF2A1	General transcription factor IIA, 1	14q24.3-q31.3	Up	2.02	0.9997	0.015
TCTEX1D2	Tctex1 domain containing 2	3q28 - q29	Up	8.51	0.9996	0.018
PDIA5	Protein disulfide isomerase family A, member 5	3q11.1 - q29	Up	35.47	0.9992	0.026
YEATS2	YEATS domain containing 2	3q25.31 - q29	Up	4.76	0.9992	0.026
PIGX	Phosphatidylinositol glycan anchor biosynthesis, class X	3q28 - q29	Up	15.51	0.9992	0.026
IKBKAP	Inhibitor of kappa light polypeptide gene enhancer in B-cells, kinase complex-associated protein	9q22.32 - q34.3	Up	9.45	0.9991	0.027
LRCH3	Leucine-rich repeats and calponin homology (CH) domain containing 3	3q28 - q29	Up	153.33	0.9990	0.028
ZNF704	Zinc finger protein 704	5q21.11 - q24.3	Up	2.96	0.9989	0.030
RFC4	Replication factor C 4	3q25.31 - q29	Up	3.59	0.9989	0.030
PAIP1	Poly(A) binding protein interacting protein 1	5p15.33 - p11	Up	3.81	0.9986	0.034
LSG1	Large subunit GTPase 1 homolog	3q28 - q29	Up	231.29	0.9984	0.036
PTP4A3	Protein tyrosine phosphatase type IVA, member 3	8q21.11 - q24.3	Up	21362.13	0.9983	0.037
C9orf72	Chromosome 9 open reading frame 72	9p23 - p21.1	Up	134.50	0.9981	0.040
DZIP3	DAZ interacting protein 3, zinc finger	3q11.1 - q29	Up	64.25	0.9981	0.040
CAMK2N2	Calcium/calmodulin-dependent protein kinase II inhibitor 2	3q25.31 - q29	Up	5.76	0.9980	0.041
EXOSC3	Exosome component 3	9p23 - p13.1	Up	3.40	0.9975	0.045
ZNF7	Zinc finger protein 7	8q21.11 - q24.3	Up	18.07	0.9971	0.048
C3orf33	Chromosome 3 open reading frame 33	3q11.1 - q29	Up	527.12	0.9980	0.049
IP6K2	Inositol hexakisphosphate kinase 2	3p26.3 - p11.1	Down	3.00	0.9999	0.004
GNRHR	Gonadotropin-releasing hormone receptor	4q11 - q25	Down	24.84	0.9998	0.011

APEH	N-acylaminoacyl-peptide hydrolase	3p26.3 - p11.1	Down	2.72	0.9998	0.013
STAP1	SH3 and cysteine rich domain	4q11 - q25	Down	4.59	0.9997	0.015
GBA3	Glucosidase, beta, acid 3 (cytosolic)	4p16.3 - p11	Down	97.96	0.9997	0.015
TMEM42	Transmembrane protein 40	3p26.3 - p11.1	Down	2.77	0.9997	0.017
WNT5A	Wingless-type MMTV integration site family, member 5A	3p26.3 - p11.1	Down	10.04	0.9996	0.018
UQCRC1	Ubiquinol-cytochrome c reductase core protein I	3p26.3 - p11.1	Down	3.44	0.9996	0.019
C3orf38	Chromosome 3 open reading frame 38	3p26.3 - p11.1	Down	16.85	0.9995	0.019
RPL29	Ribosomal protein L29	3p26.3 - p11.1	Down	4.98	0.9993	0.024
PRKAR2A	Protein kinase, cAMP-dependent, regulatory, type II, alpha	3p26.3 - p11.1	Down	3.56	0.9986	0.034
MAP4	Microtubule-associated protein 4	3p26.3 - p11.1	Down	4.53	0.9986	0.034
AZI2	5-azacytidine induced 2	3p26.3 - p11.1	Down	38.52	0.9985	0.039
ALAS1	Aminolevulinate, delta-, synthase 1	3p26.3 - p11.1	Down	4.99	0.9982	0.038
GOLGA4	Golgin A4	3p26.3 - p11.1	Down	4.53	0.9979	0.041
FBLN2	Fibulin 2	3p26.3 - p11.1	Down	144.08	0.9979	0.041
SH3TC1	SET domain and mariner transposase fusion gene	4p16.3 - p11	Down	5.35	0.9976	0.044
REST	RE1-silencing transcription factor	4q11 - q25	Down	4.35	0.9975	0.045
FBXO25	F-box protein 25	8p23.3 - p23.2	Down	3.32	0.9974	0.046
SHQ1	SH3 domain and tetratricopeptide repeats 1	3p26.3 - p11.1	Down	4.70	0.9971	0.049
ATG7	Autophagy related 7	3p26.3 - p11.1	Down	22.22	0.9970	0.049

Abbreviations: CNV-FC= Copy number associated aberration in gene expression.
